# Raman Imaging of Nanocarriers for Drug Delivery

**DOI:** 10.3390/nano9030341

**Published:** 2019-03-03

**Authors:** Sally Vanden-Hehir, William J. Tipping, Martin Lee, Valerie G. Brunton, Anna Williams, Alison N. Hulme

**Affiliations:** 1 EaStCHEM School of Chemistry, University of Edinburgh, David Brewster Road, Edinburgh EH9 3FJ, UK; s1014966@sms.ed.ac.uk (S.V.-H.); William.Tipping@ed.ac.uk (W.J.T.); 2 Edinburgh Cancer Research UK Centre, University of Edinburgh, Western General Hospital, Crewe Road South, Edinburgh EH4 2XR, UK; Martin.Lee@ed.ac.uk (M.L.); v.brunton@ed.ac.uk (V.G.B.); 3 MRC Centre for Regenerative Medicine, University of Edinburgh, Edinburgh BioQuarter, 5 Little France Drive, Edinburgh EH16 4UU, UK; anna.williams@ed.ac.uk

**Keywords:** Raman imaging, nanocarriers, drug delivery, confocal Raman, hyperspectral imaging, coherent anti-Stokes Raman scattering, stimulated Raman scattering

## Abstract

The efficacy of pharmaceutical agents can be greatly improved through nanocarrier delivery. Encapsulation of pharmaceutical agents into a nanocarrier can enhance their bioavailability and biocompatibility, whilst also facilitating targeted drug delivery to specific locations within the body. However, detailed understanding of the in vivo activity of the nanocarrier-drug conjugate is required prior to regulatory approval as a safe and effective treatment strategy. A comprehensive understanding of how nanocarriers travel to, and interact with, the intended target is required in order to optimize the dosing strategy, reduce potential off-target effects, and unwanted toxic effects. Raman spectroscopy has received much interest as a mechanism for label-free, non-invasive imaging of nanocarrier modes of action in vivo. Advanced Raman imaging techniques, including coherent anti-Stokes Raman scattering (CARS) and stimulated Raman scattering (SRS), are paving the way for rigorous evaluation of nanocarrier activity at the single-cell level. This review focuses on the development of Raman imaging techniques to study organic nanocarrier delivery in cells and tissues.

## 1. Introduction

Nanocarriers are used as drug delivery vehicles, both in research and clinical settings, because of their ability to encapsulate therapeutics and give controlled release in a biological environment [1,2,3,4]. Many potential pharmaceutics have sufficient in vitro efficacy, but fail to make it through clinical trials because of poor bioavailability [5], which nanocarrier formulations have the potential to improve. Nanocarrier-drug conjugates can improve drug solubility and can also increase the lifetime of a drug in vivo; this is especially important for protein therapeutics, which are particularly sensitive to changes in environment and are often metabolized to an inactive form before they have reached their target site [6].

The sustained release mechanism from a nanocarrier can allow the dosage of drug administered to be lowered, therefore increasing safety, and can increase time spent in the therapeutic window without the need for sequential dosing [7]. If targeted, drug delivery devices can be used to sequester drugs that have harmful off-target effects, such as cancer therapeutics, from the rest of the body [8]. Various nanocarrier targeting strategies are available, for instance, surface functionalization with antibodies, peptides, and proteins [9,10], and smart materials have been developed which release cargo upon a stimulus in vivo, for example reduced pH and hypoxia [11], both of which are found in solid tumors.

In addition to increasing the safety and activity of drugs, nanocarriers are also used to target specific areas of the body which would be otherwise difficult for the free drug to reach, for example to the central nervous system (CNS), where drugs must pass the blood-brain barrier (BBB) [12]. The BBB is a protective layer around the brain that only allows permeation of molecules that are highly lipophilic and usually less than 500 Da [13]. Many therapeutics, especially protein-based, do not fit these criteria, leading to significant efforts to deliver drugs to the brain inside nanocarriers [14,15].

A wide range of applications of nanoparticle drug delivery have been reported, and the delivery of nanoparticles using oral, intravenous, transdermal, and inhalation routes have been investigated [6]. Nose-to-brain delivery is emerging as an important field for treatment of neurological disorders, as nanocarriers can bypass the BBB in this way [16,17].

**Figure 1:**
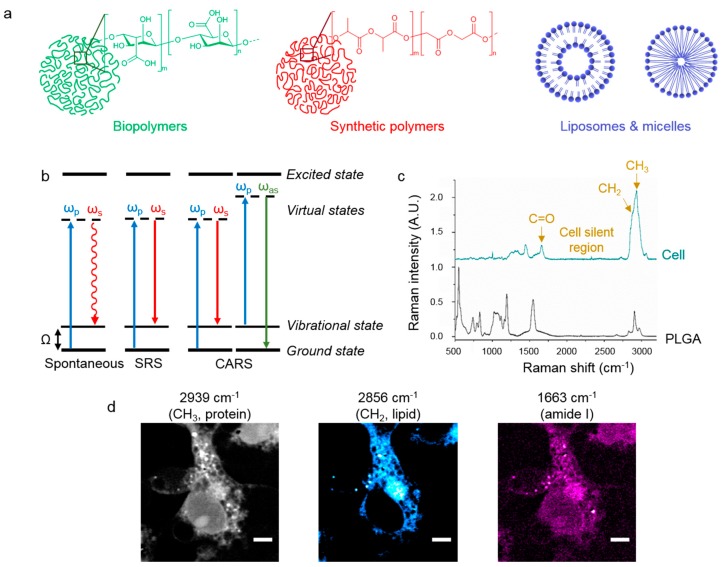
Raman imaging of nanocarriers. (**a**) Representation of different materials which can be fabricated into nanocarriers, such as biopolymers (e.g., alginate), synthetic polymers (e.g., PLGA), and lipids (as liposomes and micelles). (**b**) Energy level diagrams showing the processes of spontaneous Raman, stimulated Raman scattering (SRS), and coherent anti-Stokes Raman scattering (CARS). (**c**) Spontaneous Raman spectra showing the characteristic peaks in microglia (top, green spectrum) and PLGA, a common polymer for drug delivery (bottom, black spectrum). Spectra are normalized and offset for clarity. (**d**) SRS images of microglia when Ω = 2939 cm^−1^ (CH_3_, proteins, grey), 2856 cm^−1^ (CH_2_, lipids, cyan), and 1663 cm^−1^ (amide I, magenta). Scale bars = 5 µm.

Examples of materials used to produce nanocarriers are the natural polymers alginate and chitosan [18], synthetic polymers, such as poly lactic acid (PLA) and poly lactic-*co*-glycolic acid (PLGA) [19], and lipid-based materials, including liposomes and micelles (Figure 1a) [20]. PLGA is the most commonly used polymer for drug delivery purposes, as it has the advantage of being approved by the US Food and Drug Administration (FDA) [21]. Additionally, it is biocompatible and breaks down into lactic and glycolic acid, which are metabolized in the Krebs cycle.

There are various ways of fabricating materials into nanocarriers, depending on the desired properties of the final formulation and the drug to be encapsulated. Often, the polymer is dissolved in an organic solvent prior to emulsification with an aqueous phase to form nano-sized droplets, which become the nanocarriers upon evaporation of the organic solvent [22]. Hydrophobic drugs can be added into the organic phase with the polymer, whilst the process can be modified to a double water-in-oil-in-water emulsion to encapsulate hydrophilic drugs. Liposomes are generally formed by a lipid film hydration method [23], and micelles will self-assemble in an aqueous solution above the critical micelle concentration [24].

A critical requirement for assessing the success of nanocarriers to deliver drugs to their targeted site is the ability to image them in a biological environment. Elucidation of the mechanism of uptake, distribution, interaction, and excretion of nanoparticles is often not fully understood. Therefore, imaging techniques which offer assessment of nanoparticle drug delivery are of great importance. Previously, nanocarriers have been imaged by the encapsulation of fluorescent dyes [25,26,27], however, this has disadvantages, including loss of dye from the nanocarrier as it degrades, and photobleaching of the fluorophore. In this review we will describe efforts to image polymeric and lipid-based nanocarriers using Raman spectroscopy, which can offer label-free contrast based on molecular vibrations in the sample.

## 2. Raman Spectroscopy

Raman spectroscopy refers to the inelastic scattering of light upon interaction with a molecule, and can be used to generate a characteristic chemical fingerprint of a sample. The technique was first discovered by C.V. Raman in 1928, who observed “modified scattered radiation” when a beam of sunlight was focused on samples [28]. Raman scattering is an inherently weak effect, since most light absorbed by a molecule is elastically scattered at the same energy at which it was absorbed, in a process known as Raleigh scattering. However, if the incident photon loses or gains energy as it interacts with the molecule, this produces Stokes or anti-Stokes Raman scattering respectively.

### 2.1. Spontaneous Raman Spectroscopy

Figure 1b shows the energy level diagrams corresponding to different types of Raman spectroscopy. In spontaneous Raman, a pump laser is used to excite the sample and the inelastic scattering of photons is detected, usually at the Stokes-shift, which gives a stronger signal as more molecules populate the ground state at ambient conditions. Plotting the resultant Raman intensity against the Raman shift in wavenumbers gives rise to Raman spectra. As an example, we have shown the Raman spectrum of microglia, which are glial cells that act as phagocytes in the brain (Figure 1c). Importantly, this spectrum shows no peaks between 1800 and 2800 cm^−1^, known as the cell-silent region. This region can be exploited with chemical tags to allow imaging of nanocarriers against the cellular background signals [29], as discussed in Section 3.3. In contrast to infrared spectroscopy, which is also used as a biomedical imaging technique [30], Raman spectroscopy has a decreased sensitivity to water making it an ideal technique to probe biological samples.

### 2.2. Increasing Raman Sensitivity

Raman scattering is a weak process, with only 1 in every 10^8^ molecules being inelastically scattered [31]. The cross-section of spontaneous Raman scattering is extremely small compared to fluorescence [32], which can limit the speed of acquisition of biological images by Raman spectroscopy [33]. This has led to various methods being developed to amplify the Raman signal produced.

#### 2.2.1. Surface Enhanced Raman Scattering (SERS)

Surface enhanced Raman scattering (SERS) involves the interaction of a Raman reporter with a roughened metal surface [34], which gives an electromagnetic enhancement of signal in the order of 10^4^ to 10^8^ over spontaneous Raman [35]. It has been used for lipid bilayer characterization [35], intracellular redox potential sensing [36], and pathogen diagnosis [37].

#### 2.2.2. Resonance Raman Scattering

Resonance Raman scattering occurs when the frequency of the incident laser matches, or is close to, the frequency of an electronic transition of the target sample, which results in scattering enhancements in the region of 10^3^ to 10^4^ over spontaneous Raman [38,39]. Improvements to sensitivity and selectivity are achieved because only the selected chromophore is in resonance, and thus generates the amplified signal. The technique has been used to detect antioxidants using iron oxide nanoparticles [40], and to achieve live cell organelle imaging [41].

#### 2.2.3. Coherent Raman

Coherent Raman techniques use two incident laser beams with a frequency difference tuned to a vibration of interest [42,43]. This drives molecules with a matching vibration into resonance with one another, and the emitted waves are phase matched, allowing them to interfere constructively. This gives a signal increase over spontaneous Raman but is limited to a single vibrational mode. However, multiple incident laser beams can drive responses at multiple vibrational frequencies. Unlike SERS, a metal is not required for signal enhancement, and coherent Raman can generate high resolution images, such that sub-cellular features can be readily identified [43].

Coherent Raman was first demonstrated for biological imaging as coherent anti-Stokes Raman spectroscopy (CARS), where the incoming pump beam (ω_p_) and Stokes beam (ω_S_) interact with the sample in a four wave mixing process, generating a strong anti-Stokes signal at ω_aS_ = 2 ω_p_ − ω_S_ (Figure 1b) [44]. CARS was first demonstrated by Maker and Terhune in 1965 for analyzing molecular vibrational spectra of gases [45], however, it was not until 1999 that it was adapted into an imaging modality for biological samples [46]. CARS imaging offers high spatial resolution with contrast derived from the inherent chemical bonds of the sample [47]. Additionally, CARS imaging can achieve fast optical sectioning to generate 3D-resolved stacks and images with depth penetration reported into the 100 µm range [48]. Recently, Fourier-transform CARS has been used to achieve high-throughput sorting of two different types of polymer beads, and also live cells on a microfluidic chip [49]. Although CARS signal is enhanced when Δω matches a molecular vibration, it also occurs in non-resonant conditions, leading to high background levels and a complex relationship between CARS and spontaneous Raman spectra.

Many of the issues of CARS are averted in stimulated Raman scattering (SRS) [50,51], which measures the stimulated emission as either an intensity loss in the pump beam (stimulated Raman loss, SRL) or as an intensity gain in the Stokes beam (stimulated Raman gain, SRG). When Δω does not match a molecular vibration within the sample, SRL and SRG cannot occur. As a result, there is no associated non-resonant background, rendering image analysis much simpler than in CARS imaging. SRS also has the advantage over CARS that it replicates the spontaneous Raman spectrum, and permits quantitative detection [52]. In comparison to spontaneous Raman imaging, SRS provides a 10^8^ enhancement in excitation efficiency [53], and over 1000-fold improvement in image acquisition speed through stimulated emission of the vibration mode of interest [54].

In Figure 1d we have shown representative images of microglia imaged by SRS microscopy, which are the same cells that have been analyzed by spontaneous Raman in Figure 1c. Tuning Ω to the large CH_3_ peak at 2939 cm^−1^ produces a label-free image indicative of the cellular protein content. Similarly, tuning Ω to 2856 cm^−1^ builds up a strong signal from the CH_2_ content which is present in membranous structures in the cell and notably absent from the nucleus. The amide I peak at 1663 cm^−1^ also shows the cellular proteins, but with a weaker signal than the CH_3_ peak.

## 3. Confocal Raman Imaging

Many of the studies using Raman to image nanocarriers have utilized confocal Raman microscopy, where the Raman laser is interfaced to an optical microscope. The use of confocal Raman for biological applications has recently been reviewed by Gomes da Costa et al., who give examples of mapping compounds within cells and tissues in a non-destructive manner by Raman microscopy [55]. The imaging set-up uses a single laser at fixed wavelength to excite Raman-active modes in the sample. Taking a Raman spectrum at each pixel in the sample allows a distribution map of chemical species, such as proteins and lipids, which can be assigned to different organelles to be built up. This allows a label-free image of the cell to be acquired without the use of any dyes as required for fluorescence imaging.

### 3.1. Analysis of Hyperspectral Images

When spontaneous Raman spectroscopy is coupled with the spatial power of an imaging technique, a large amount of data is generated. Hyperspectral Raman imaging, also known as Raman mapping, involves raster scanning across a sample and taking a Raman spectrum at each pixel; it has been used to image plaques in the brains of Alzheimer’s patients [56], and for the diagnosis of cancer [57,58,59].

The complex raw data acquired from the Raman spectrometer may be processed to distinguish small changes in spectroscopic peaks in order to de-convolute overlapping substances in each pixel, for example proteins and lipids. Since Raman spectra involve measurements at hundreds of different wavenumbers, and contain spectral information of multiple chemical species, multivariate statistical analysis can be utilized [60]. Two common methods are principal component analysis (PCA) and vertex component analysis (VCA). PCA reduces the spectral information in each pixel by finding the independent spectra which hold the most variation in Raman signal [61]. VCA does not consider the variation, but defines the outer space of observed spectra [62]. Both methods allow the decomposition of each pixel’s spectrum into main spectral components. Plotting the color-coded components can then be used to extract hidden, or known, features inside the image, such as proteins and lipids.

The spontaneous Raman spectrum of PLGA is provided in Figure 1c. PLGA is the most widely used polymer for drug delivery and has been used extensively in Raman studies. There are characteristic peaks for CH_2_ (2876 cm^−1^), CH_3_ (2949 cm^−1^), and carbonyl (C=O, 1766 cm^−1^) bonds, but there are no peaks in the cell-silent region. Chemometric techniques (PCA and VCA) are important when imaging nanocarriers where the polymeric peaks overlap with cellular peaks, for example in the fingerprint region (500–1700 cm^−1^) of PLGA.

**Figure 2:**
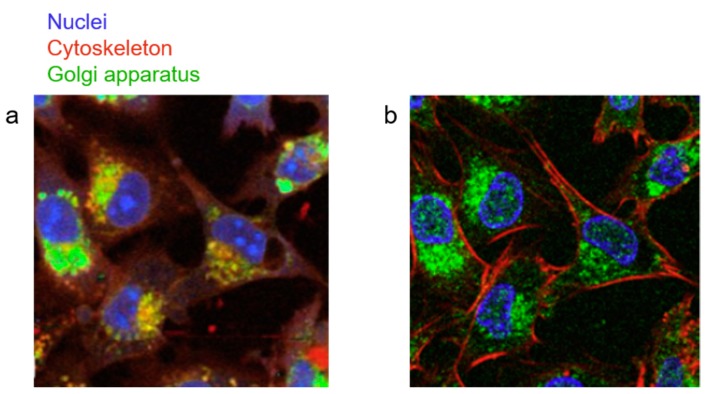
Comparison of Raman-based, artificial IF and fluorescence imaging. (**a**) A Raman-based, artificial IF image of cultured human glioma cells with false color assignments showing the nuclei in blue, cytoskeleton in red, and Golgi apparatus in green. (**b**) An immunofluorescent image of the same cells staining the nuclei (DAPI, blue), cytoskeleton (rhodamine-conjugated phalloidin, red), and Golgi apparatus (anti-Syntaxin-6-antibody, green). Reproduced with permission from [63], Copyright Elsevier, 2012.

Since fluorescence imaging is the gold-standard in many laboratories, Klein et al. used both immunofluorescence (IF) staining and confocal Raman imaging to probe the nucleus, cytoskeleton, and Golgi apparatus of the same cells [63]. Raman is non-destructive, so these images were acquired before staining the cells with fluorescent markers. PCA was used to identify the main components of the Raman image, which were assigned to organelles according to their similarity to their IF counterparts to predict Raman-based, artificial IF cells. A key advantage of Raman is that more than three organelles can be identified from the label-free Raman signal, and it is not limited by overlapping Stokes’ shifts of fluorophores, as in IF. Figure 2 shows that there is good overlap between the Raman-based artificial IF (a) and IF (b) images, and that similar features are observed in both.

### 3.2. Label-Free Imaging of Drug Delivery

In addition to imaging cellular components, confocal Raman microscopy has also been used to investigate drug delivery to cells by nanocarriers. Doxorubicin is a clinical chemotherapeutic agent which is widely used to treat a variety of different cancers through inhibition of topoisomerase-II, which stalls cell replication [64]. Due to its short life in vivo and the fact that the molecule is inherently fluorescent, doxorubicin has become a popular model drug for nanocarrier delivery systems.

Romero et al. encapsulated doxorubicin inside PLGA nanoparticles using the double-emulsion method and studied their effect on the human HepG2 cell line [65]. Taking advantage of the fluorescent properties of doxorubicin, they used flow cytometry to evaluate the uptake into cells, and viability assays confirmed that doxorubicin still exerted the desired chemotherapeutic effect. In addition, the group used confocal Raman to confirm the DNA-chelation mode of action of doxorubicin by studying the Raman fingerprint of the DNA. A DNA/protein ratio was calculated and it was observed that in doxorubicin treated cells, this ratio was lower compared to the control cells. This is indicative of DNA damage in the doxorubicin treated group.

The same authors also produced PLGA nanoparticles surface-coated with an antiTNF-α antibody layer for delivery to HepG2 cells. Confocal Raman was used to confirm the co-localization of the nanoparticle, antiTNF-α, and cell cytoplasm by their unique Raman signals [66]. Although all three components have overlapping Raman spectra with no labels in the cell-silent region, there are characteristic peaks visible for the PLGA, antibody, and cytoplasm when a Raman spectrum is taken at a single location on the treated HepG2 cells.

Finally, Romero et al. also used confocal Raman imaging to study the intracellular location of PLGA nanoparticles in HepG2 cells and found them to co-localize with lipid droplets identified in the CH_2_ image [67].

### 3.3. Imaging with Bioorthogonal Labels

One of the main advantages of Raman spectroscopy over traditional fluorescence imaging is that it is a label-free technique. However, recently, small chemical tags have been developed as a way to enhance contrast. This topic was reviewed in 2017 by Zhao et al. [68]. There are no peaks in the Raman spectrum of a cell between 1800 and 2800 cm^−1^, and various bond stretches, such as carbon–deuterium (C–D), alkynes (C≡C), and nitriles (C≡N) generate peaks in this region, meaning they can be imaged without interference from the cellular background [69]. Alkyne tags have been shown to have a strong Raman vibrational cross-section and allow imaging in a bio-orthogonal manner. A number of alkyne-tagged molecules have been used to probe biological processes, such as DNA, RNA and protein synthesis [70,71,72,73], glucose metabolism [74,75], and intracellular drug uptake [76,77].

### 3.4. Imaging Intracellular Uptake of Nanocarriers

An important aspect of nanocarriers is their ability to enter a cell and deliver their cargo, therefore it is critical to assess their cellular location. Depending on their size, zeta potential, and surface properties, nanocarriers can be internalized in cells by phagocytosis, endocytosis, or macro-pinocytosis [78,79]. Studying the rate of this internalization and the location of the nanocarriers in the cell is important for inferring their function as drug carriers.

#### 3.4.1. Uptake of Polymeric Nanocarriers

**Figure 3:**
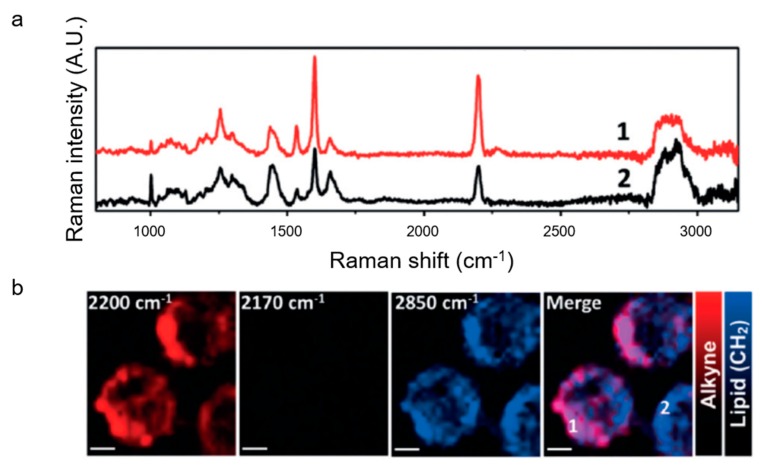
Raman imaging of poly(phenylene ethynylene) PPE nanoparticles in HeLa cells. (**a**) Spontaneous Raman spectra of two representative HeLa cells that have been incubated with TAT-PPE nanoparticles. (**b**) Raman imaging shows that the alkyne-labelled nanoparticles are inside the cytoplasm. Scale bars = 5 µm. Reproduced with permission from [80], Copyright John Wiley & Sons, 2017.

Recently, alkyne tags have also been used to label nanocarriers in order to study their intracellular behavior. In 2017, Li et al. reported Raman imaging of nanocarriers synthesized from poly(phenylene ethynylene) (PPE), a water soluble polymer which contains an intrinsic alkyne [80]. A nanoprecipitation method was used to form nanoparticles from the polymer, and the particles were also conjugated to TAT peptide, a peptide sequence known to be cell penetrant [81]. When incubated with HeLa cells, the nanoparticles were observed by Raman imaging to localize in the cytoplasm (Figure 3). The spontaneous Raman spectra in Figure 3a show a strong, sharp alkyne peak at 2200 cm^−1^, along with the characteristic cellular peaks. Figure 3b shows that the alkyne signal co-localizes with the cellular lipids (2850 cm^−1^) and that there is an absence of signal in the cell-silent region at 2170 cm^−1^, confirming that the signal in the on-resonance image is due to TAT-PPE nanoparticles.

In addition to labelling the polymer with a Raman-active tag, another strategy for imaging is to use a Raman-active payload inside the nanocarrier. This strategy has been used by two groups to investigate the uptake of polymer nanocarriers.

**Figure 4:**
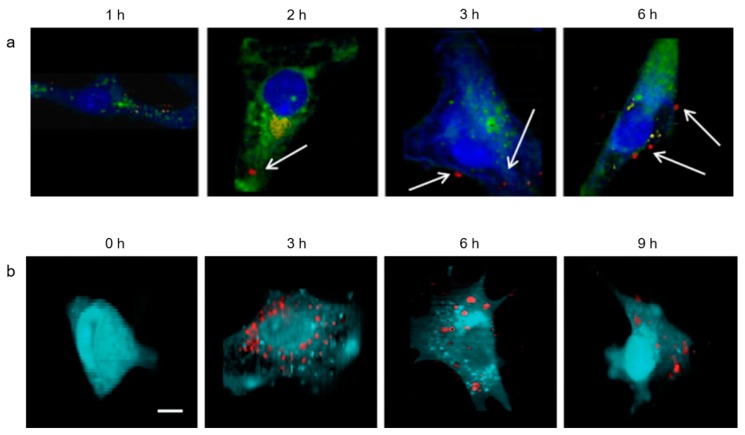
The uptake of polymeric nanocarriers. (**a**) The uptake of epidermal growth factor receptor targeted nanoparticles to SKOV-3 cells over time shows that particles enter after 2 h. Images are overlays of the cell body and nucleus (blue), membranous organelles (green), early endocytic vesicles (yellow), and nanoparticles (red). White arrows show regions of nanoparticle aggregation. Reproduced with permission from [82], published by Springer Nature (2013). (**b**) The uptake of β-carotene loaded poly lactic-*co*-glycolic acid (PLGA) nanoparticles into murine NIH-3T3 cells showing the cell body (cyan) and nanoparticles (red). Scale bar = 10 µm. Reproduced with permission from [83], Copyright John Wiley and Sons, 2013.

Chernenko et al. studied the uptake of epidermal growth factor receptor (EGFR) targeted and non-targeted polycaprolactone-PLGA nanoparticles loaded with the deuterated drug C6-ceramide-d_11_ in SKOV-3 ovarian cells [82]. Applying vertex component analysis to their hyperspectral data allowed them to visualize the cell body and nucleus (blue), membranous organelles (green), early endocytic vesicles (yellow), and the C–D stretch unique to the nanoparticles (red). These components were overlaid to show the distribution of the nanoparticles within the cell (Figure 4a). The non-targeted nanoparticles were not seen inside the cells until 6 h, whereas the EGFR targeted particles (shown in Figure 4a) were seen to enter the cells after 2 h.

In another study, β-carotene was encapsulated inside PLGA nanoparticles, which were incubated with murine NIH-3T3 cells [83]. β-Carotene has an extended vibrationally-active structure, which means that resonance Raman spectroscopy is possible at a discrete wavenumber corresponding to the conjugated double bonds in β-carotene. Comparing the spontaneous spectra of β-carotene and PLGA shows that there are strong bands unique to the β-carotene at 1520 and 1153 cm^−1^. Figure 4b shows representative images at 0, 3, 6, and 9 h incubation, with the cell body shown in cyan and the nanoparticles in red.

#### 3.4.2. Imaging Lipid-Based Nanocarriers

**Figure 5:**
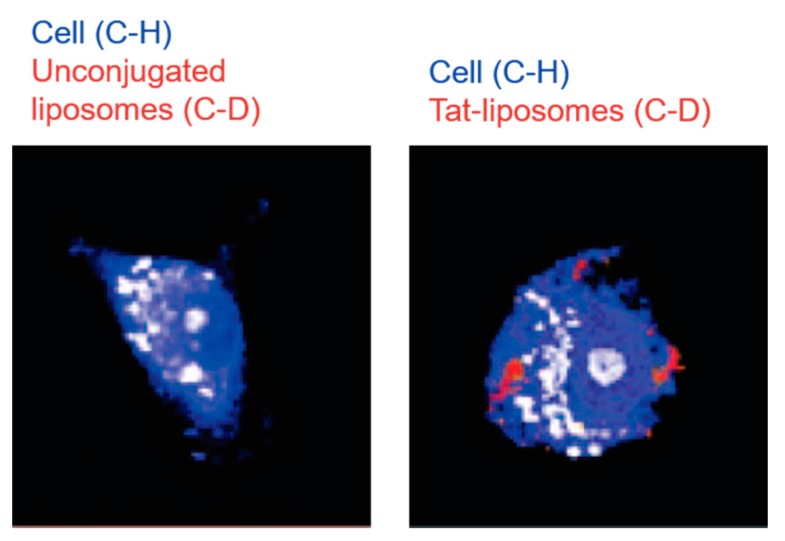
Uptake of deuterated liposomes to MCF-7 cells after 6 h of incubation. C–H stretching shown in blue and C–D stretching shown in red. There are no unlabeled liposomes inside the cell after 6 h but the TAT-liposomes are visible in the cytoplasm. Reproduced with permission from [85], Copyright American Chemical Society, 2008.

Liposomes have been used for some time as drug delivery vehicles [84]. Matthäus et al. used deuterated DSPC-d_70_ liposomes to form nanocarriers that could be tracked by Raman spectroscopy, as the C-D bonds generate Raman bands in the cell-silent region [85]. They also conjugated these liposomes to the cell-penetrant TAT-peptide and studied the difference in uptake between TAT-labelled liposomes and unconjugated liposomes in human MCF-7 cells. It was observed that while the uptake mechanisms for both were similar, there was a stark difference in the time taken for uptake to occur, with the TAT-liposomes visible after 6 h and the blank liposomes not visible until 12 h. Figure 5 shows representative cells from the 6 h time point with the C–H stretch shown in blue and the C–D stretch in red. The C–D stretch shows the location of the liposomal carriers in the cells, and it can be seen to be absent in cells treated with unconjugated liposomes at the 6 h time point.

Chernenko et al. used Raman microscopy coupled with VCA to image cationic liposomes in HeLa cells and observed intracellular cationic liposomes after 1 h, whilst natural liposomes did not enter until 12 h [86]. Raman spectroscopy has also been used to investigate the aggregation behavior of sodium dodecyl sulfate micelles [87]. It was found that pure micelles were stable but they aggregated upon the addition of ligands. Aggregation behavior is important when considering the suitability of a drug delivery system.

These studies, and the studies with polymeric carriers, show that Raman microscopy is an attractive imaging platform for monitoring nanocarrier uptake into a variety of cell types.

### 3.5. Imaging Intracellular Degradation of Nanocarriers

**Figure 6:**
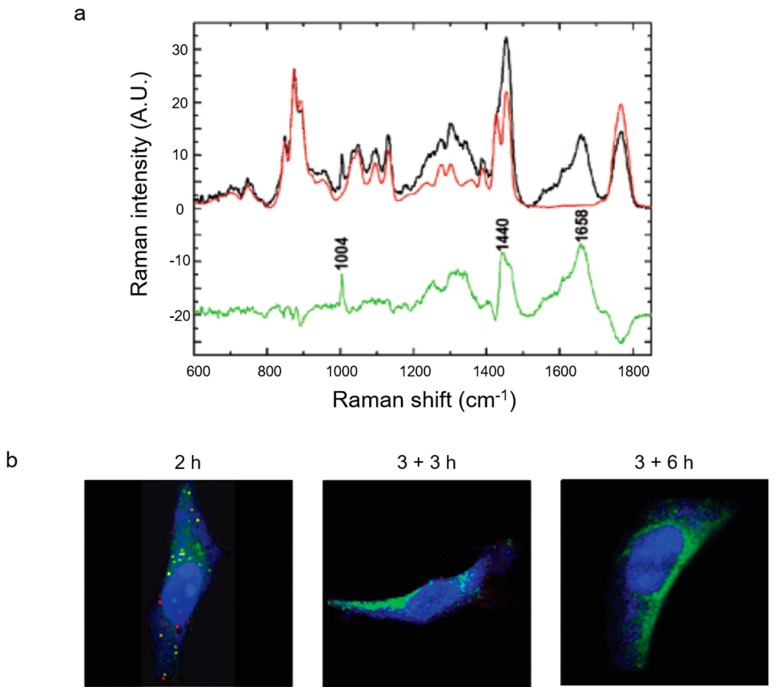
Imaging nanocarrier behavior over time. (**a**) The degradation of PLGA nanoparticles in macrophages over two weeks. Red spectrum shows pure, untreated PLGA, black spectrum shows PLGA after two weeks of degradation and the green spectrum shows the difference between the two. Reproduced with permission from [88], Copyright American Chemical Society, 2004. (**b**) PLGA nanoparticles incubated with HeLa cells showing proteins in blue, lipids in green, and nanoparticles in red. After 2 h of incubation with nanoparticles they were visible by Raman. After 3 h incubation with nanoparticles then 3 or 6 h of incubation with fresh media, nanoparticles were no longer visible. Reproduced with permission from [89], Copyright American Chemical Society, 2009.

In addition to following nanocarrier internalization into cells, it is also important to study the degradation behavior of nanocarriers over time, since most polymeric carriers hydrolyze in an aqueous environment to release their cargo. In the case of PLGA, water molecules hydrolyze the polymer’s ester bonds. Pioneering work by van Apeldoorn et al. in 2004 monitored the loss of intensity of PLGA ester bonds at 1768 cm^−1^, corresponding to degradation of the polymer (Figure 6a) [88]. The spectrum in red shows pure PLGA which has not undergone any degradation, and the black spectrum is of the PLGA nanoparticles which have been allowed to degrade inside macrophages for two weeks. After normalizing both of these spectra to the C–COO stretch of lactic acid at 875 cm^−1^, the green line shows the difference between the two, with a clear reduction in the carbonyl band at 1768 cm^−1^. This corresponds to a reduction in the number of ester bonds of the PLGA. As the PLGA degrades, there is also an increase in cellular peaks, such as phenylalanine at 1004 cm^−1^, lipids at 1440 cm^−1^, and the amide I band at 1658 cm^−1^, corresponding to ingress into the particle.

Chernenko et al. used Raman imaging coupled with VCA to image HeLa cells incubated with PLGA nanoparticles [89]. Figure 6b shows the cellular protein in blue, lipids in green, and PLGA aggregates in red. They observed nanoparticles present in the cells after 2 h. Another group of cells were incubated with the particles for 3 h, before changing the media to remove extracellular particles and leaving for either a further 3 or 6 h. As shown in Figure 6b, there are no nanoparticles present in either of these cases, inferring their degradation or exocytosis.

## 4. Coherent Raman Imaging

### 4.1. In Vitro Imaging

**Figure 7:**
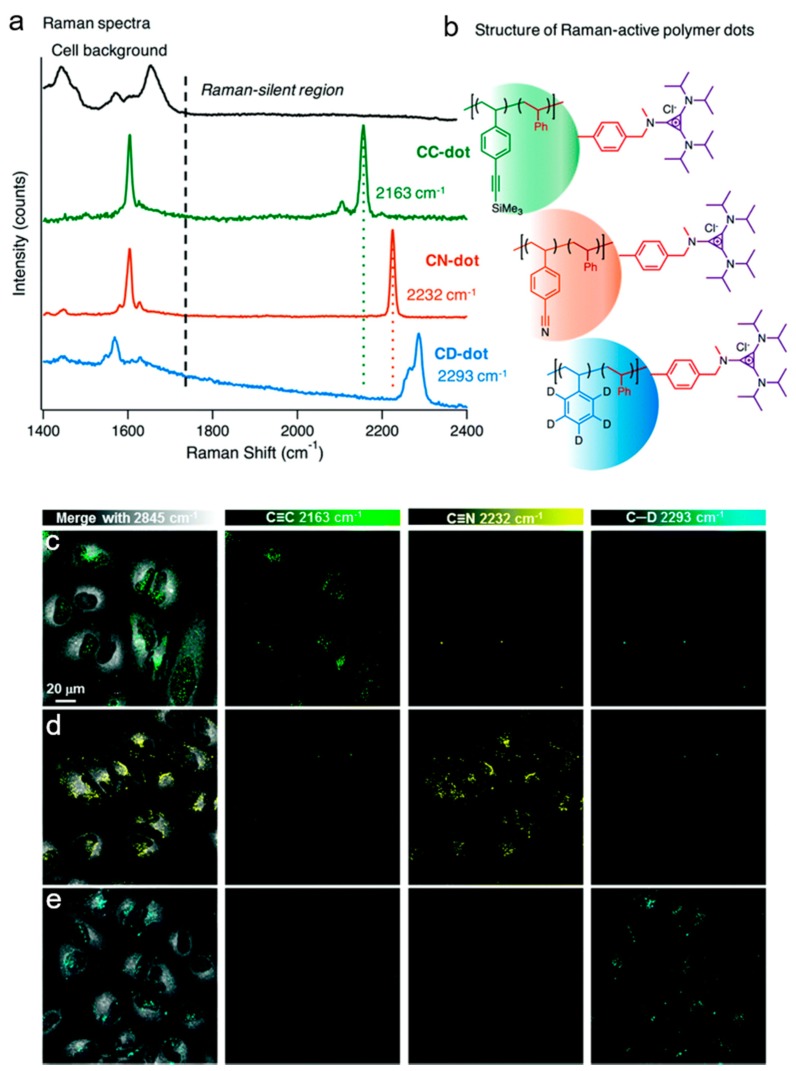
Analysis of Raman-active polymer dots using spontaneous Raman spectroscopy and SRS imaging. (**a**) Raman spectra of nanoparticles with orthogonal groups in the cell silent region (1800–2800 cm^−1^). (**b**) Corresponding structures of the Raman-active polymer dots, comprising of a core of styrene and styrenic derivatives (C≡C, C≡N and C–D labelled styrene) and surface-coated with trisaminocyclopropenium (TAC) groups. (**c**–**e**) SRS imaging of Raman-active polymer dots in live HeLa cells. The polymer dots are detected at discrete frequencies in the HeLa cells: (**c**) C≡C dot (2163 cm^−1^), (**d**) C≡N dot (2232 cm^−1^), and (**e**) C–D dot (2293 cm^−1^), with the cellular contrast detected at 2845 cm^−1^ (CH_2_, lipids) in the merge images. Reproduced with permission from [90], Copyright Royal Society of Chemistry, 2017.

SRS imaging is an emerging technique for evaluating nanoparticle dynamics at the cellular level. Recently, Hu et al. demonstrated that SRS imaging could be used in tandem with bio-orthogonal labelling of styrene-based nanoparticles, to enable multiplex detection of three Raman-active nanoparticle species in living HeLa cells [90]. Raman-active monomers incorporating alkyne, nitrile, and C–D groups were used to prepare Raman-active polymer dots (Figure 7). The cellular uptake of each of the nanoparticles into live HeLa cells within 2 h was confirmed using SRS imaging (Figure 7). Multiplex detection of the three Raman-active nanoparticles was achieved in a co-culture of COS-7, MEF, and HeLa cells, pre-treated with alkyne (2163 cm^−1^), nitrile (2232 cm^−1^), and C-D (2293 cm^−1^) nanoparticles, respectively. This study demonstrated the ability to monitor the uptake kinetics, distribution, and intracellular photostability of the nanoparticles. As such, SRS imaging of nanoparticles in this way is anticipated to have far-reaching opportunities for live-cell imaging in biotechnology and theranostic applications.

Hyperspectral SRS imaging has been developed to enable chemical fingerprinting of a sample by sweeping the laser wavelength to generate an SRS spectrum. It has been used to study the uptake of metallic nanoparticles into *T. thermophilia* cells (unicellular free-living eukaryotic ciliate) [91]. The study demonstrated the use of hyperspectral SRS imaging to determine the uptake routes and target sites of two different inorganic nanoparticle species inside the cells, and assessed the competitive uptake of polyacrylate (PAA)-coated hematite (α-Fe_2_O_3_) and anatase (TiO_2_). Combined, these studies indicate the potential to study nanoparticle dynamics at the cellular level.

### 4.2. Coherent Raman Imaging of Nanoparticle Interactions Ex Vivo

#### 4.2.1. Drug Delivery to the Skin

Topical drug delivery is an effective treatment strategy for diseases of the skin and nail because the active formulation is locally applied to the affected area. Direct targeting in this way is often a preferred therapeutic strategy for the delivery of the active pharmaceutical agent because it has the potential to reduce first-pass metabolism of the drug, which is associated with systemic oral delivery [92]. Consequently, the active concentration of drug can be maximized at the required site. The impermeable nature of the skin renders transdermal drug delivery a major challenge when developing novel pharmaceutic agents to treat infections of the skin [93].

**Figure 8:**
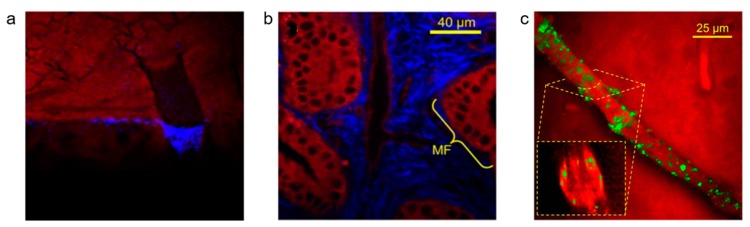
Multimodal imaging of nanoparticle delivery in tissue. (**a**) Porated skin incubated with deuterated methyl methacrylate nanoparticles. Image contrast derived from coherent anti-Stokes Raman scattering (CH_2_, 2855 cm^−1^, red) and stimulated Raman scattering (CD_2_ nanoparticles, 2120 cm^−1^, blue). Reproduced with permission from [98], Copyright Elsevier, 2014; (**b**) Planar cross section through a sample of mouse gall bladder. A mucosal fold (MF) is indicated. Image contrast derived from two photon fluorescence (endogenous fluorophores, red) and second harmonic generation (collagen, blue). Reproduced with permission from [102], Copyright John Wiley and Sons, 2012; (**c**) Cross-section of a mouse-brain blood vessel following incubation with deuterated quaternary ammonium palmitoyl glycol chitosan nanoparticles. Image contrast derived from epi-detection CARS microscopy of lipid (CH_2_, 2845 cm^−1^, red) and nanoparticles (C–D, 2100 cm^−1^, green). Reproduced with permission from [107]. Copyright Springer Nature, 2016.

SRS microscopy has been widely used to study the architecture of the skin [94,95], whilst the penetration of pharmaceutically relevant solvents, for example water, DMSO/DMSO-d_6_, and deuterated propylene glycol have been studied in skin [96] and nail samples [97]. CARS and SRS microscopy have been used together to study the distribution of deuterated methyl methacrylate nanoparticles in porated skin samples [98]. CARS imaging at 2855 cm^−1^ (CH_2_, lipid) was used to generate contrast of the skin surface, while SRS microscopy was used at 2120 cm^−1^ (CD_2_) to investigate the distribution of the deuterated nanoparticles (Figure 8a).

In summary, SRS and CARS microscopy have advanced current understanding within dermato-pharmacokinetics, enabling visualization of the architecture and morphology of the skin using intact skin samples. Non-invasive imaging offers a significant improvement to conventional tape-stripping experiments, which aim to investigate the penetration of small-molecules and nanocarriers by sequentially revealing the outer-most layer of the skin and using chemical analysis to determine the local concentration within each strip [99]. Additionally, SRS imaging may enable visualization of slow-release formulations to the skin (and other organs); a recent example demonstrated SRS imaging of the dissolution of entecavir, a hepatitis B antiviral drug, embedded in a slow release PLA formulation, as a potential step towards this aim [100]. Thus, it is anticipated that SRS and CARS microscopy will help to improve the mechanistic understanding of nanoparticle-based dermal formulations.

#### 4.2.2. Drug Delivery to Targeted Organs

The application of CARS imaging to study nanoparticle uptake within a number of biological organs has been demonstrated. Oral delivery of deuterated biopolymer-based quaternary ammonium palmitoyl glycol chitosan (dGCPQ) nanoparticles has been studied using CARS microscopy in the liver [101], kidney [32], and the gastrointestinal tract [102]. Epi-detection CARS imaging at ~2845 cm^−1^ (C–H, tissue) and 2100 cm^−1^ (C–D nanoparticles) was used to provide contrast [103]. Additionally, a multimodal imaging approach using second harmonic generation (SHG), two photon fluorescence (TPF), and CARS has enabled detailed analysis of the in vivo dynamics of the nanoparticles (Figure 8b). As a result, a more complete assessment of the interactions of nanoparticles at the tissue level could be achieved through multimodal imaging.

#### 4.2.3. Drug Delivery to the Brain

The delivery of drugs and therapeutic peptides to the brain is an active area of research, due to the difficulty of penetrating the BBB and rapid in vivo metabolism of peptide drugs [104]. Significant advances in delivering peptides to the central nervous system have been realized using polymer nanoparticles to deliver therapeutic peptides to the brain following intranasal administration [105], and using a nanofiber-based delivery strategy [106]. More recently, dGCPQ nanoparticles were administered intravenously to live mice for the delivery of lomustine, a small-molecule drug used in glioblastoma treatment [107]. The nanoparticles were visualized in ex vivo brain samples, and were found to be located within blood vessels in the brain slice (Figure 8c). The nanoparticles were not detected in the brain parenchyma, but were adhered to the BBB, where it was reasoned that the drug payload was released for transport across the BBB. These studies demonstrate the versatility of coherent Raman imaging techniques to provide mechanistic understanding of the uptake, delivery, and fate of nanoparticles in a variety of cells, tissues, and organs within the body.

## 5. Conclusions and Future Perspectives

Raman imaging has become a valuable and established analytical technique for the study of organic nanocarriers in vitro and in vivo. These nanocarriers are made of a wide variety of materials from natural and synthetic polymers to lipid-based materials. Polymeric and liposomal carriers are extremely popular for clinical uses due to their biocompatible properties [108]. Since PLGA is FDA approved, this makes it another attractive clinical material for the future. However, as the clinical potential for nanocarriers is realized, concerns regarding their toxicity also become prominent [109].

Raman is non-destructive, and unlike fluorescence imaging, does not rely on bulky labels for visualization. Nanocarriers have been imaged in a wide variety of cells and tissues either in a label-free manner, utilizing multivariate statistics for deconvolution, or with small chemical tags, such as carbon-deuterium isotopologues, or alkyne bonds. A major advantage of Raman is that it allows direct imaging of the nanocarriers, and not the payload encapsulated within them. This is especially important when studying the uptake and degradation of nanocarriers; factors which have been shown to be vital for inferring their likely success as intracellular drug delivery vehicles. Unsurprisingly, the type of cell used seems to make a significant difference on nanocarrier behavior, with a variety of uptake and degradation times reported. These times will also vary depending on the size, surface properties, and zeta-potential of the nanocarrier.

Coherent Raman imaging has been shown to increase the contrast of Raman images to visualize nanoparticles in tissues. Most recently, stimulated Raman scattering has been used to provide multi-color images of nanoparticle behavior in cells. We believe that the signal enhancement provided by SRS, coupled with the ability to attach small chemical labels to nanocarriers, indicates the potential of SRS to have profound influence in future studies on nanocarrier dynamics at the cellular level. As Raman imaging moves towards clinical applications, future opportunities for imaging the distribution of nanocarriers in living systems in real-time may be realized.
